# miR-506 attenuates methylation of lncRNA *MEG3* to inhibit migration and invasion of breast cancer cell lines via targeting SP1 and SP3

**DOI:** 10.1186/s12935-018-0642-8

**Published:** 2018-10-25

**Authors:** Xin-Xing Wang, Guang-Cheng Guo, Xue-Ke Qian, Dong-Wei Dou, Zhe Zhang, Xiao-Dong Xu, Xin Duan, Xin-Hong Pei

**Affiliations:** Department of Breast Surgery, the First Affiliated Hospital of Zhengzhou University, No.1, East Jianshe Road, Erqi District, Zhengzhou, 450052 Henan People’s Republic of China

**Keywords:** Breast cancer, Migration and invasion, *miR*-*506*, SP3, DNMT1, *MEG3*, Methylation

## Abstract

**Background:**

Breast cancer has been the first death cause of cancer in women all over the world. Metastasis is believed to be the most important process for treating breast cancer. There is evidence that lncRNA *MEG3* functions as a tumor suppressor in breast cancer metastasis. However, upstream regulation of *MEG3* in breast cancer remain elusive. Therefore, it is critical to elucidate the underlying mechanism upstream MEG3 to regulate breast cancer metastasis.

**Methods:**

We employed RT-qPCR and Western blot to examine expression level of *miR*-*506*, DNMT1, SP1, SP3 and *MEG3*. Besides, methylation-specific PCR was used to determine the methylation level of *MEG3* promoter. Wound healing assay and transwell invasion assay were utilized to measure migration and invasion ability of breast cancer cells, respectively.

**Results:**

SP was upregulated while *miR*-*506* and *MEG3* were downregulated in breast tumor tissue compared to adjacent normal breast tissues. In addition, we found that *miR*-*506* regulated DNMT1 expression in an SP1/SP3-dependent manner, which reduced methylation level of *MEG3* promoter and upregulated *MEG3* expression. SP3 knockdown or *miR*-*506* mimic suppressed migration and invasion of MCF-7 and MDA-MB-231 cells whereas overexpression of SP3 compromised miR-506-inhibited migration and invasion.

**Conclusions:**

Our data reveal a novel axis of *miR*-*506*/SP3/SP1/DNMT1/*MEG3* in regulating migration and invasion of breast cancer cell lines, which provide rationales for developing effective therapies to treating metastatic breast cancers.

## Background

Breast cancer is the most common cause of cancer-related death in women worldwide [1–3]. Similar to other solid tumors, distant metastasis (especially lung metastasis) is the leading cause of breast cancer-associated death and resistance to various treatments [4]. Therefore, it is essential for us to elucidate the molecular mechanism underlying breast cancer progression.

MicroRNAs are around 22-nucleotide-long non-coding RNAs [5]. They modulate gene expression through targeting 3′-UTR of mRNAs, leading to mRNA degradation or translational block [6, 7]. Many studies have revealed that miRNAs are involved in cell growth, migration, apoptosis and cancer metastasis [8–10]. Arora et al. [11] reported that *miR*-*506* had a role in regulating EMT in breast cancer cell lines. As a validation, Yu et al. [12] has shown that *miR*-*506* overexpression inhibits proliferation and metastasis of breast cancer cells. However, the mechanism of *miR*-*506* inhibition of breast cancer metastasis remains elusive.

*MEG3* is identified as an imprinted gene with maternal expression and encodes a long non-coding RNA [13]. Dysregulation of *MEG3* has been found in various human tumors, including bladder cancer, hepatocellular carcinoma, lung cancer and ovarian cancer [14–16]. More interestingly, *MEG3* has been implicated into tumorigenesis and progression of breast cancer [17, 18]. Previous studies have revealed that overexpression of *MEG3* could induce cell growth arrest and increase cell apoptosis in human breast cancer cells. In addition, downregulated *MEG3* regulates proliferation, migration and invasion of breast cancer in a p53-dependent manner [17]. Whether *miR*-*506* cooperates with *MEG3* to regulate the metastasis of breast cancer remains unclear. In pituitary tumors, hypermethylation of the *MEG3* regulatory region is identified as an important mechanism associated with the loss of *MEG3* expression [19]. *miR*-*29a* was shown to regulate methylation of *MEG3* via DNA methyltransferase (DNMT) 1 and 3b, thus contributing to hepatocellular carcinoma (HCC) growth [20]. Likewise, Li et al. [18] demonstrated that *MEG3* was epigenetically repressed by DNMT1 to suppress the p53 pathway in glioma. Based on these findings, we hypothesized that *MEG3* may be regulated in a DNA methylation-dependent manner in breast cancer cells.

SP1 and SP3 transcription factors are expressed in almost all mammalian cells. They belong to the specificity protein/Kruppel-like factor (SP/KLF) transcription factor family and are involved in regulation of DNMTs [21]. Davie et al. [22] showed SP1 and SP3 could either enhance or repress the activity of promoters of genes implicated in differentiation, cell cycle progression, and oncogenesis. Although SP1 and SP3 has been investigated in breast cancer, the detailed mechanism by which SP1 and SP3 regulate progression of breast cancer requires to be further investigated [23].

Here, we show that *miR*-*506* inhibits migration and invasion of breast cancer cell lines through the SP3/DNMT1/*MEG3* axis. Our findings reveal the detailed mechanism by which *miR*-*506* regulates metastasis of breast cancer, which facilitates the development of therapeutical strategies for treating breast cancer.

## Materials and methods

### Patients and samples

The present study was approved by the Ethics Committee of The First Affiliated Hospital of Zhengzhou University. A total of 20 breast tumor samples and 20 adjacent normal tissue samples were obtained from patients aged 20–70 in 2016–2017. No patients had received chemotherapy or radiotherapy prior to surgery. Breast cancer was validated by histological examination in all cases according to World Health Organization criteria. Breast tumors and normal tissue specimens excised surgically from patients were immediately snap-frozen and stored in liquid nitrogen until use.

### Cell lines

Human breast cancer cells (MCF-7, MDA-MB-231, SKBR3) and Human Embryonic Kidney (HEK) 293T cells were purchased from ATCC and cultured in Dulbecco’s Modified Eagle’s Medium (DMEM, Hyclone) supplemented with 10% fetal bovine serum and 100 U/ml penicillin/streptomycin at 37 °C, 5% CO_2_. Human breast epithelial MCF10A cells were grown in the base medium for this cell line (MEBM) along with the appropriate additives (MEGM, Lonza/Clonetics Corporation, CC-3150). HEK 293T cells were employed in lentiviruses packaging.

### Plasmid generation and lentivirus package

SP3 cDNA was cloned into pcDNA4 vector. The short hairpin RNA (shRNAs) targeting SP3 (target sequence showed blow) were purchased from GenePharma, Shanghai, China and cloned into PLKO.1 vector. To generate lentiviruses, the packaging vectors (pPAX2 and pVSVG) and transfer vector were co-transfected into 293T cells. The supernatant was harvested at 24 h and 48 h after transfection and filtered through 0.45 μm membrane. For virus infection, the virus supernatant was added to medium at 1:3 ratio, 24 h later, 2ug/ml puromycin was used to select stable cell lines. scramble shRNA and empty pcDNA4 vector was used as negative control respectively.

shRNA targeting sequences of SP3: shSP3#1: GCAAGAACTGTGGTGTCTTGG; shSP3#2: CCTTCTGCTAACATCCAGAAT; shSP3#3: CGCGAGATGATACTTTGATTA; shSP3#4: GTGGTGATTCTACCTTGAATA.

### Transfection

For NC and *miR*-*506* mimic transfection, we used LipofectamineVR LTX with PlusTM Reagent (Life Technologies) according to manufacturer’s instructions. mimic NC and miR-506 mimics were synthesized by GenePharma.

### Wound healing assay

Migration of cells was measured by a wound healing assay in vitro. Briefly, 2 × 10^5^ MCF-7 and MDA-MB-231 cells were seeded onto 6-well plates, and incubated in appropriate complete culture medium for 16 h under normal conditions at 37 °C. The monolayer was scratched and incubated in fresh medium without FBS for 24 h. Finally, the wound width was measured. Three different locations were visualized and photographed under inverted microscope.

### Invasion assay

Invasion assays was performed using chambers containing 8.0-μm pore membranes (Millipore) with matrigel basement membrane matrix. Breast cancer cells (1 × 10^5^ cells) were resuspended in 200 µl of FBS-free medium, and then seeded into the top chamber with Matrigel-coated membrane. Next, 500 µl medium with 10% FBS was added to the bottom chamber as a chemoattractant. After 48 h of incubation, the non-invaded cells were removed from the upper surface of the membrane with a cotton-tipped swab, and the invaded cells were fixed, stained with crystal violet, finally, 5 fields of the stained cells per sample were counted under the inverted microscope.

### Western blot

The cells were harvested and lysed by RIPA buffer. The lysates were boiled at 100 °C for 5 min and centrifuged at 10,000 rpm for 1 min. About 50 ug of total protein were loaded onto SDS-PAGE gel. After that, the proteins were transferred to PVDF membrane at 300 mA for 2.5 h. The membrane was blocked with 5% non-fat milk in 1 × TBST for 1 h at room temperature, then incubated with primary antibodies at 4 °C overnight. Then, the membrane was washed with 1 × TBST for 3 times, 5 min each time, and incubated with secondary antibodies at room temperature for 1 h. Finally, the membrane was incubated with ECL and exposed. The following antibodies were used: anti-SP3 (Santa Cruz, USA), anti-DNMT1 (Cell Signaling Technology, USA), anti-SP1 (Cell Signaling Technology, USA), anti-β-actin (Proteintech).

### RT-qPCR

Cells were harvested and RNA was extracted by Trizol method, then chloroform was added to the mixture. The sample was centrifuged at 12,000 rpm for 10 min and transferred to new RNase-free EP tubes, mixed with an equal volume of isopropanol and centrifuged. Supernatant was then removed and 75% ethanol added to wash the pellet. Finally, ethanol was discarded and the pellet dried and resuspended in 20–30 µl Rnase-free H_2_O.

For reverse transcription, ~ 1 ug of total RNA was used for reverse transcription according to manufacturer’s instruction (TAKARA PrimeScript Kit).

For real time PCR, we used SYBR as a probe dye to detect the signal, with GAPDH used as internal control. The Ct value was calculated using the ^ΔΔ^Ct method and normalized to GAPDH levels. The following primers were used:

- *MiR*-*506*-QPCR-F: GCCACCACCATCAGCCATAC
- *MiR*-*506*-QPCR-R: GCACATTACTCTACTCAGAAGGG
- *MEG3*-QPCR-F: ATCATCCGTCCACCTCCTTGTCTTC
- *MEG3*-QPCR-R: GTATGAGCATAGCAAAGGTCAGGGC
- DNMT1-QPCR-F: CGGCTTCAGCACCTCATTTG
- DNMT1-QPCR-R: AGGTCGAGTCGGAATTGCTC
- SP1-QPCR-F: CTGGTCCCATCATCATCCGG
- SP1-QPCR-R: TGTTTGGGCTTGTGGGTTCT
- SP3-QPCR-F: GGTCAAGTCCAGGTTCAGGG
- SP3-QPCR-R: CTGAGAACTGCCCGAGAGTC
- GAPDH-QPCR-F: GAGTCAACGGATTTGGTCGT
- GAPDH-QPCR-R: TTGATTTTGGAGGGATCTCG

### Luciferase assay

The 3′-UTRs of SP1 and SP3 were fused to the luciferase gene using the XhoI/NotI restriction sites in the psiCHECK2vector. Mutations in the miR-506 target site in these UTRs were generated using the QuikChange Multi Site-directed Mutagenesis kit (Stratagene, LaJolla, CA). Luciferase assays were performed using the Dual-Luciferase assay (Promega). Renilla expression was normalized to the luciferase gene on the psiCHECK2 vector.

### Methylation-specific PCR

DNA methylation status was examined by the methylation-specific PCR with genomic DNA treated with sodium bisulfite using the EZ DNA MethylationDirect kit (Zymo Research). Two primer sets were used to amplify the promoter region of the *MEG3* gene containing a number of CpG sites, one for the methylated sequence (forward, 5′-TATGAGTTGTAAGCGGTAGAGTTC-3′; reverse, 5′-TACGAACTTAACGAAAAAAAATCAT-3′) and the other for the unmethylated sequence (forward, 5′-GAATATGAGTTGTAAGTGGTAGAGTTT-3′; reverse, 5′-TACAAACTTAACAAAAAAAAATCATACT-3′).

### Statistical analysis

Each experiment was performed for three times independently. All values were presented as mean ± SD. Comparisons were performed using student’s *t*-test for two groups or one-way ANOVA for multiple groups. *P < 0.05 was considered statistically significant.

## Results

### SP3 depletion suppresses migration and invasion of breast cancer cells

**Fig. 1:**
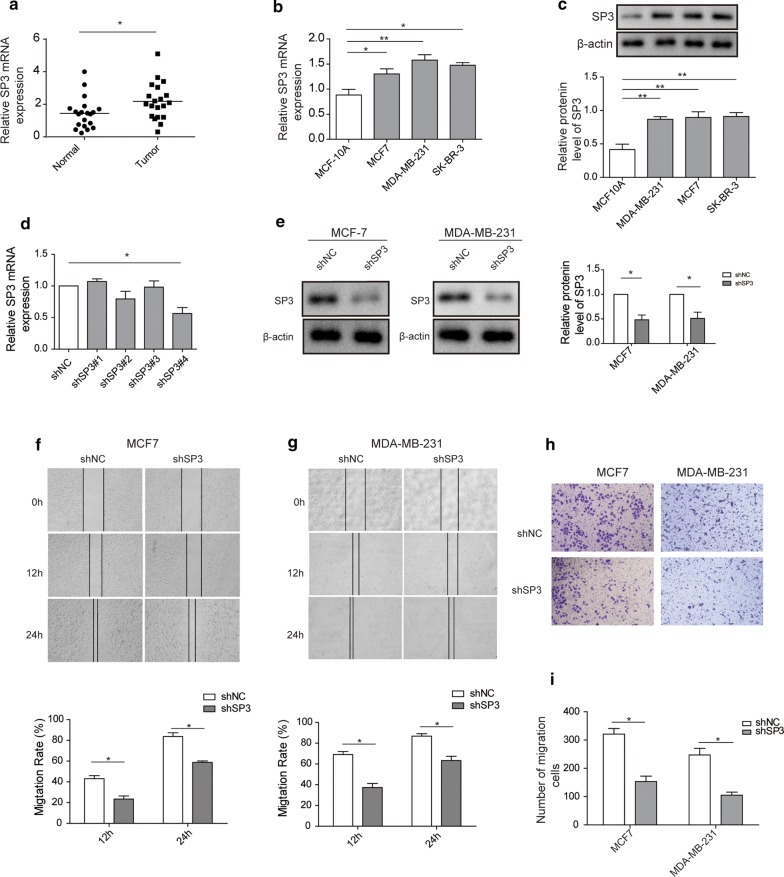
SP3 depletion suppresses migration and invasion of breast cancer cells. **a** RT-qPCR analysis showing the mRNA level of SP3 in normal breast tissues and breast tumors. GAPDH acts as internal control. **b** RT-qPCR analysis showing the mRNA level of SP3 in MCF10A and breast cancer cell lines (MCF-7, MDA-MB-231, SKBR3). GAPDH acts as internal control. **c** Western blot analysis showing the protein level of SP3 in MCF10A and breast cancer cell lines (MCF-7, MDA-MB-231, SKBR3). β-actin acts as internal control. **d** RT-qPCR analysis showing the mRNA level of SP3 in MCF-7 transfected with shSP3#1, shSP3#2, shSP3#3, shSP3#4. **e** Western blot analysis and quantification showing the protein level of SP3 in MCF-7 or MDA-MB-231 cells transfected with shNC or shSP3(shSP3#4). β-actin acts as internal control. **f** Wound healing assay and quantification showing migration of shNC or SP3 knockdown MCF-7 cells. The photographs were taken at time points of 0, 12, 24 h. **g** Wound healing assay and quantification showing migration of shNC or SP3 knockdown MDA-MB-231 cells. The photographs were taken at time points of 0, 12, 24 h. **h** Transwell invasion assay and quantification showing the invasion ability of shNC or SP3 knockdown MCF-7 cells. 5 fields were counted per sample and the photographs were taken at 24 h. **i** Transwell invasion assay and quantification showing the invasion ability of shNC or SP3 knockdown MDA-MB-231 cells. 5 fields were counted per sample and the photographs were taken at 24 h. All data were presented as mean ± SD from three biological replicates (*P < 0.05; **P < 0.01)

We firstly attempted to examine the effect of SP3 on metastasis of breast cancer. We probed the mRNA level of SP3 in normal and breast tumor tissues and observed that expression of SP3 was upregulated in breast tumors (n = 20) relative to normal breast tissues (n = 20) (Fig. 1a). Next, we employed RT-qPCR and Western blot to detect mRNA and protein level of SP3 in normal and breast cancer cell lines, respectively. In agreement with results in tissues, expression levels of SP3 were higher in breast cancer cells (MDA-MB-231, MCF-7 and SKBR3) compared to MCF10A (Fig. 1b, c).

Next, we sought to explore the influence of SP3 on migration and invasion of MCF-7 and MDA-MB-231 cells. We examined the knockdown efficiency of 4 designed shRNAs against SP3 and found that shSP3#4 was the validated one at both mRNA and protein levels (Fig. 1d, e). Next, we performed wound healing assay to evaluate migration ability of human breast cancer cells with or without SP3. The results revealed that SP3 knockdown markedly reduced the migration ability of MCF-7 and MDA-MB-231 cells (Fig. 1f, g). Besides, we assessed whether SP3 had a role in the invasion ability of MCF-7 and MDA-MB-231 cells by transwell invasion assay. Expectedly, SP3 depletion suppressed invasion of the two breast cancer cells (the invaded cell number was decreased by ~ 50% in SP3-silenced cells) (Fig. 1h, i).

### SP3 knockdown downregulates DNMT1 expression and decreases methylation level of *MEG3* promoter

**Fig. 2:**
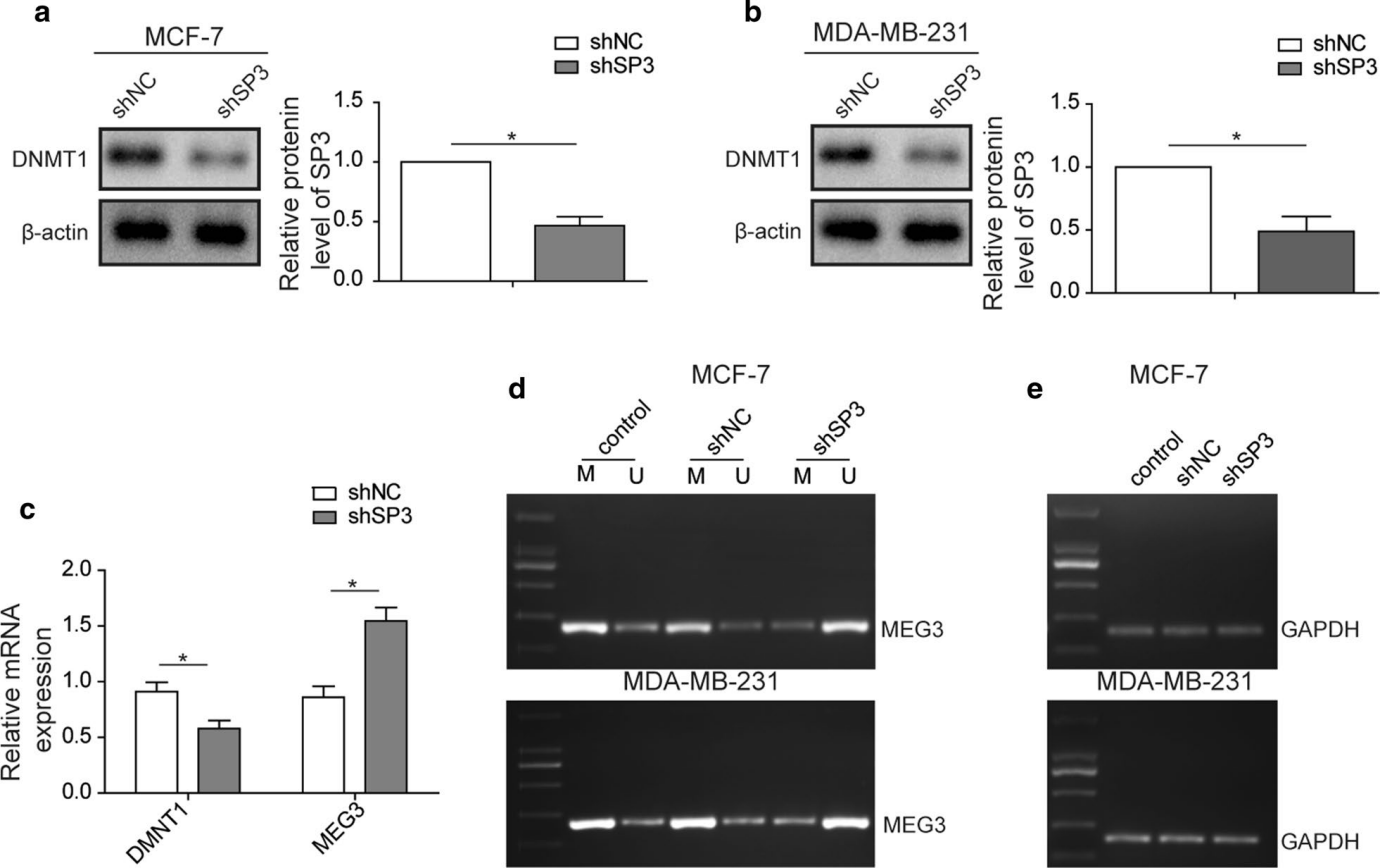
SP3 knockdown downregulates DNMT1 expression and decreases methylation level of MEG3 promoter. **a** Western blot analysis showing the protein level of DNMT1 and SP3 in shNC or SP3 knockdown MCF-7 cells. β-actin acts as internal control. **b** Western blot analysis showing the protein level of DNMT1 in shNC or shSP3 transfected MDA-MB-231 cells. β-actin acts as internal control. **c** RT-qPCR analysis showing the mRNA level of DNMT1 and MEG3 in shNC or SP3 knockdown MDA-MB-231 cells. GAPDH acts as internal control. **d** Methylation-specific PCR (MSP) analysis showing the methylation level of MEG3 promoter in wild-type (control), shNC or SP3 knockdown MCF-7 cells. GAPDH acts as negative control. **e** Methylation-specific PCR (MSP) analysis showing the methylation level of MEG3 promoter in wild-type (control), shNC or SP3 knockdown MDA-MB-231 cells. GAPDH acts as negative control. All data were presented as mean ± SD from three biological replicates (*P < 0.05)

SP3 cooperates with p300 to control DNMT1 expression [24]. To explore the mechanism underlying SP3 knockdown-attenuated metastasis of MCF-7 and MDA-MB-231 cells, we attempted to confirm whether SP3 also impacted the expression of DNMT1 in breast cancer cells. Consistent with previous reports, SP3 knockdown led to decrease in DNMT1 expression in MCF-7 and MDA-MB-231 cells (Fig. 2a, b). Furthermore, we found that SP3 silencing decreased DNMT1 levels but also upregulated *MEG3* (Fig. 2c). As DNMT1 has been known as a DNA methyltransferase, we hypothesized that SP3 knockdown reduced DNMT1 levels, which in turn altered *MEG3* methylation level and increased *MEG3* expression. To validate this hypothesis, we carried out methylation-specific PCR to examine *MEG3* methylation level and observed that, in both MCF-7 and MDA-MB-231 cells, SP3 knockdown substantially decreased the methylation level of *MEG3*, whereas unmethylated *MEG3* levels was increased in SP3-depleted cells (Fig. 2d, e). Taken together, SP3 silencing could result in decreased expression of DNMT1 and then reduce methylation level of *MEG3*, finally thereby increasing *MEG3* levels.

### Reduced expression of *miR*-*506* and *MEG3* in breast tumors and cell lines

**Fig. 3:**
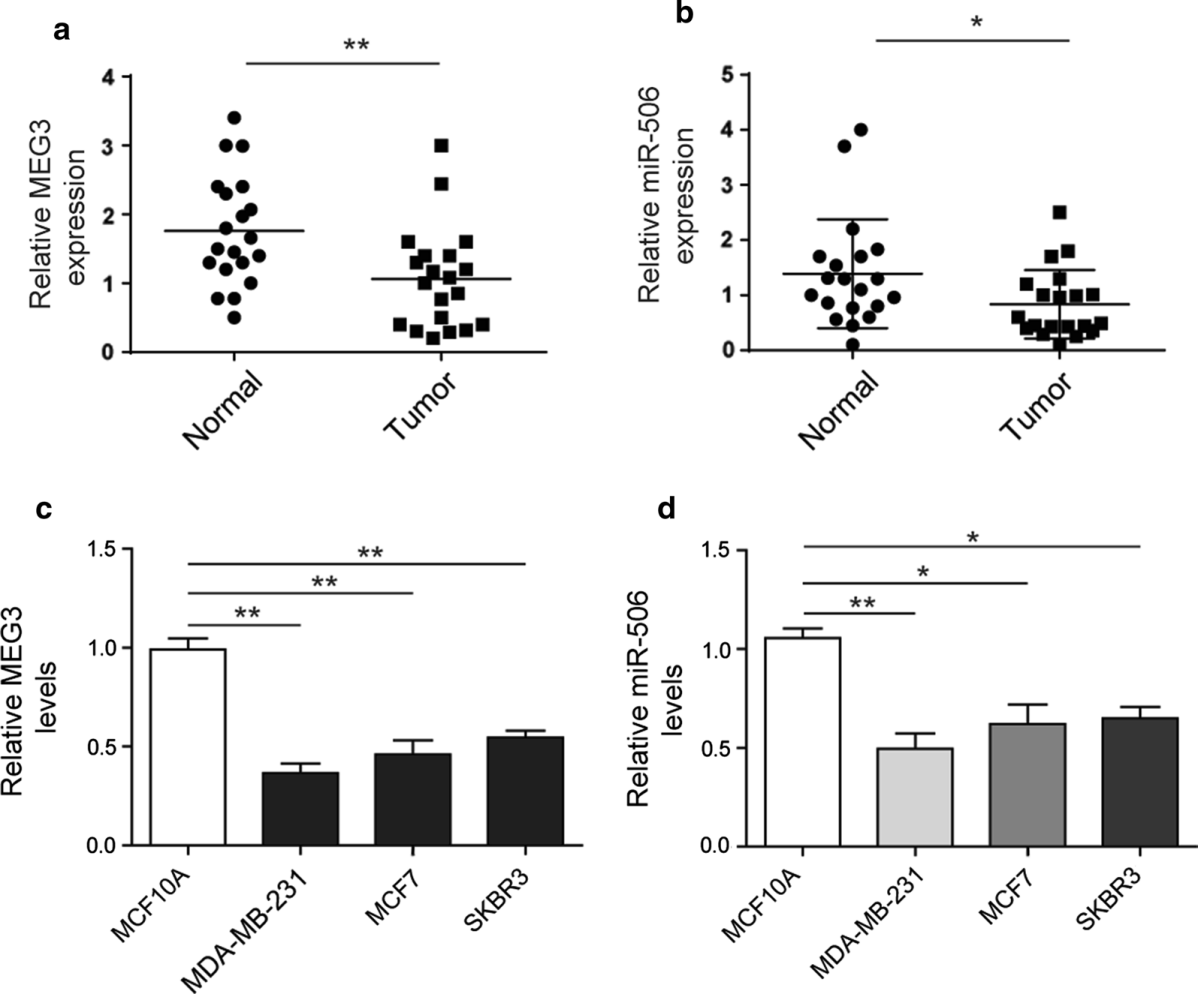
Reduced expression of miR-506 and MEG3 in breast tumors and cell lines. **a** RT-qPCR analysis showing the level of miR-506 in normal breast tissues and breast tumors. GAPDH acts as internal control. **b** RT-qPCR analysis showing the level of MEG3 in normal breast tissues and breast tumors. GAPDH acts as internal control. **c** RT-qPCR analysis showing the level of miR-506 in MCF10A and breast cancer cell lines (MCF-7, MDA-MB-231, SKBR3). GAPDH acts as internal control. **d** RT-qPCR analysis showing the level of MEG3 in MCF10A and breast cancer cell lines (MCF-7, MDA-MB-231, SKBR3). GAPDH acts as internal control. All data were presented as mean ± SD from three biological replicates (*P < 0.05; **P < 0.01)

To elucidate the clinical role of *miR*-*506* and *MEG3* in breast cancer progression, we determined the expression level of these two genes in normal (n = 20) and breast tumor tissues (n = 20), as well as in normal human breast epithelial cells and three breast cancer cell lines. The RT-qPCR results showed that *miR*-*506* and *MEG3* levels were significantly lower (about half) in breast tumor tissues than in adjacent normal tissues (Fig. 3a, b). In addition, we found that the levels of *miR*-*506* and *MEG3* were remarkably decreased (~ 50%) in breast cancer cell lines (MDA-MB-231, MCF-7 and SKBR3) compared to immortalized human breast epithelial cell (MCF10A) (Fig. 3c, d). These data indicated that dysregulation of *miR*-*506* and *MEG3* might be involved in breast cancer.

### *miR*-*506* overexpression downregulates SP1, SP3, DNMT1 and upregulates *MEG3* expression

**Fig. 4:**
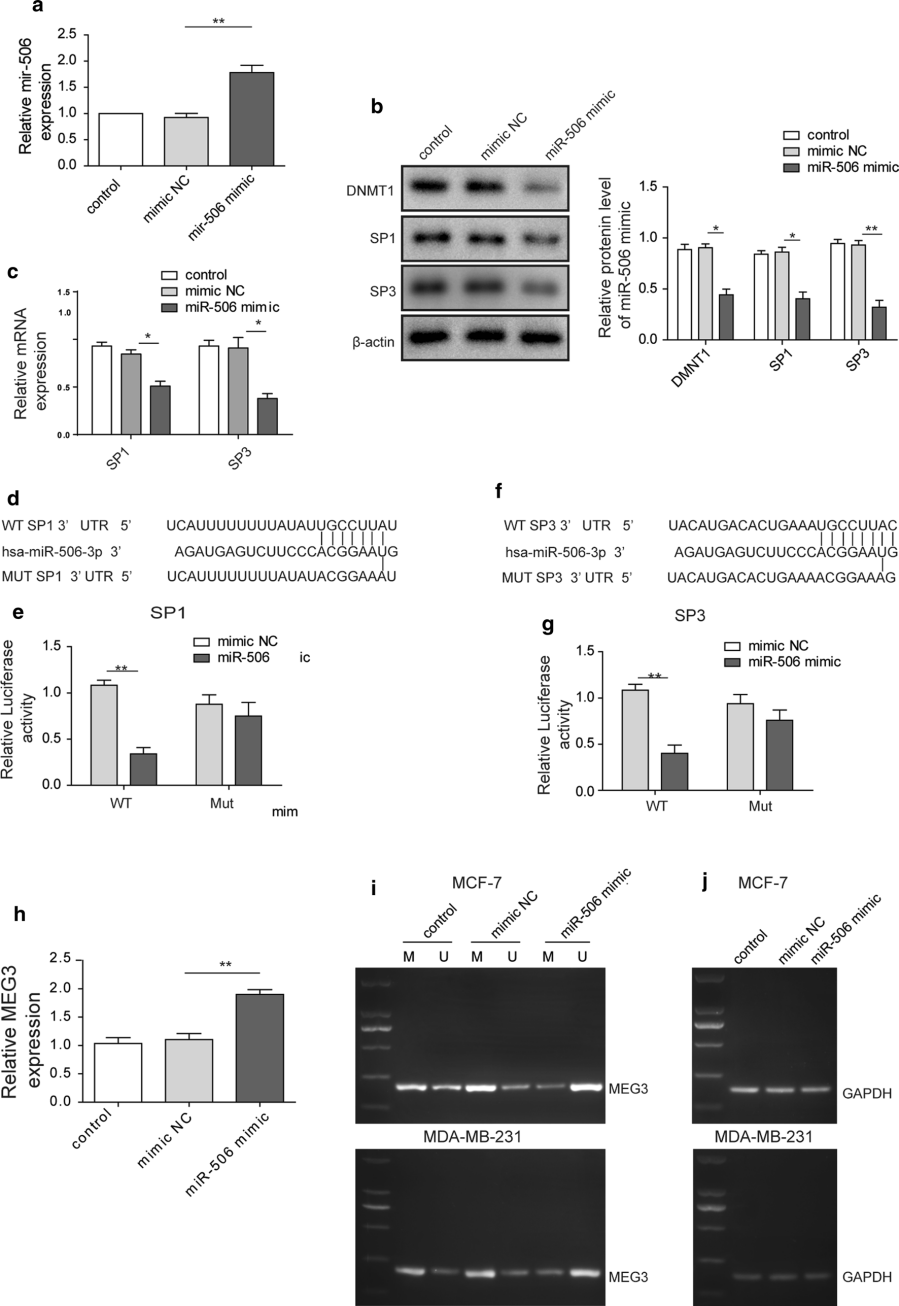
miR-506 overexpression downregulates SP1, SP3, DNMT1 and upregulates MEG3 expression. **a** RT-qPCR analysis showing the level of miR-506 in control (wild-type), mimic NC- or miR-506 mimic transfected MCF-7 cells. **b** Western blot analysis showing the protein level of DNMT1 and SP3 in control (wild-type), mimic NC- or miR-506 mimic-overexpressed MCF-7 cells. β-actin acts as internal control. **c** RT-qPCR analysis showing the mRNA level of DNMT1 and SP3 in control (wild-type), mimic NC- or miR-506 mimic-overexpressed MCF-7 cells. GAPDH acts as internal control. **d** Bioinformatic prediction of binding site at 3′-UTR of SP1 by miR-506. **e** Luciferase reporter assay showing miR-506 binds wild-type 3′-UTR of SP1, not mutant 3′-UTR of SP1. The relative luciferase activity was measured and the data were presented as mean ± SD. **f** Bioinformatic prediction of binding site at 3′-UTR of SP3 by miR-506. **g** Luciferase reporter assay showing miR-506 binds wild-type 3′-UTR of SP3, not mutant 3′-UTR of SP3. The relative luciferase activity was measured and the data were presented as mean ± SD. **h** RT-qPCR analysis showing the level of MEG3 in control (wild-type), mimic NC- or miR-506 mimic-overexpressed MCF-7 cells. GAPDH acts as internal control. **i** Methylation-specific PCR (MSP) analysis showing the methylation level of MEG3 promoter in wild-type (control), mimic NC- or miR-506 mimic-overexpressed MCF-7 cells. GAPDH acts as negative control. **j** Methylation-specific PCR (MSP) analysis showing the methylation level of MEG3 promoter in wild-type (control), mimic NC- or miR-506 mimic-overexpressed MDA-MB-231 cells. GAPDH acts as negative control. All data were presented as mean ± SD from three biological replicates (*P < 0.05; **P < 0.01)

To clarify the mechanism by which *miR*-*506* played a tumor suppressive role in migration and invasion of breast cancer cell lines, we transfected MDA-MB-231 with miR-506 mimic to overexpress miR-506 (Fig. 4a). The Western blot analysis demonstrated that *miR*-*506* overexpression led to remarkably decreased expression of DNMT1, SP1 and SP3 compared to NC mimic group (Fig. 4b). In parallel, the RT-qPCR assay revealed that expression levels of SP1 and SP3 were declined in *miR*-*506* overexpressed breast cancer cells relative to that in NC mimic cells (Fig. 4c). Since microRNAs function to regulate gene expression by targeting 3′-UTR region of mRNAs, we hypothesized that *miR*-*506* was likely to bind 3′-UTR of SP1 and SP3. The bioinformatic prediction revealed that *miR*-*506* could target 3′-UTR of SP1 and SP3 by complementary sequences, which was confirmed by luciferase assay. The results showed that *miR*-*506* only caused significant decrease (by ~ 60%) in wild-type 3′-UTR-fused luciferase reporter, not mutant 3′-UTR-fused luciferase reporter (Fig. 3d–g).

Finally, we also observed that *MEG3* level was upregulated (~ twofold) by *miR*-*506* mimic compared to NC mimic (Fig. 4h). Moreover, we utilized methylation-specific PCR to measure methylation level of *MEG3* upon miR-506 overexpression. The data demonstrated that *MEG3* methylation level decreased in MCF-7 and MDA-MB-231 cells transfected with *miR*-*506* mimic, whereas unmethylated *MEG3* was higher (Fig. 4i, j).

### SP3 compromises *miR*-*506* overexpression-attenuated migration and invasion of breast cancer cells

**Fig. 5:**
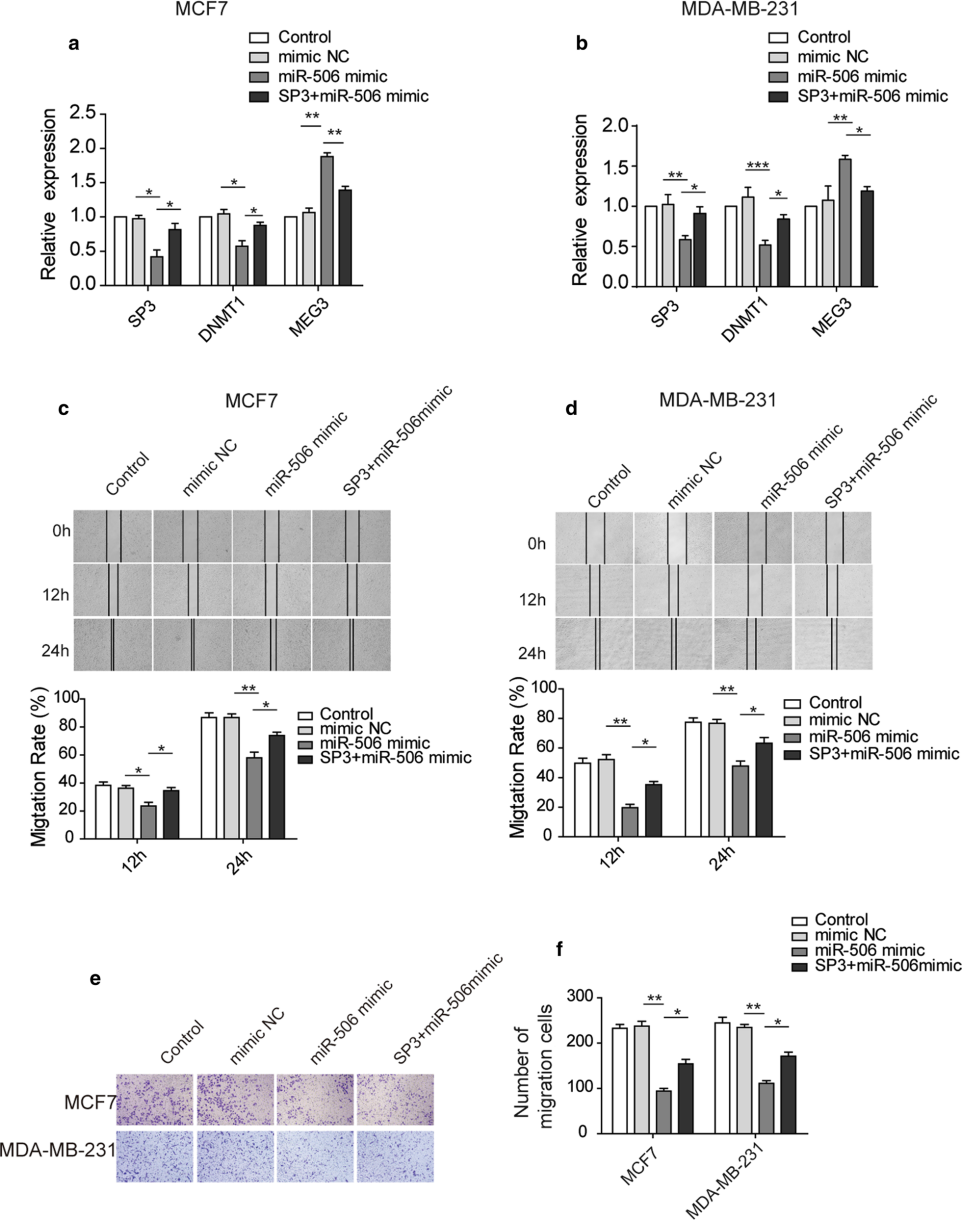
SP3 rescues miR-506 overexpression-attenuated migration and invasion of breast cancer cells. **a**, **b** RT-qPCR analysis showing the mRNA level of SP3, DNMT1 and MEG3 in control (wild-type), mimic NC-, miR-506 mimic-, miR-506 mimic plus SP3 overexpressed MDA-MB-231 and MCF-7 cells. **c** Wound healing assay and quantification showing migration of MCF-7 cells transfected with mimic NC, miR-506 mimic, SP3 plus miR-506. The images were taken at time points of 0, 12, 24 h. **d** Wound healing assay and quantification showing migration of MDA-MB-231 cells transfected with mimic NC, miR-506 mimic, SP3 plus miR-506. The images were taken at time points of 0, 12, 24 h. **e** Transwell invasion assay and quantification showing migration of MCF-7 cells transfected with mimic NC, miR-506 mimic, SP3 plus miR-506. 5 fields were counted per sample and the images were taken at time points of 0, 12, 24 h. **f** Transwell invasion assay and quantification showing migration of MDA-MB-231 cells transfected with mimic NC, miR-506 mimic, SP3 plus miR-506. 5 fields were counted per sample and the images were taken at time points of 0, 12, 24 h. All data were presented as mean ± SD from three biological replicates (*P < 0.05; **P < 0.01)

Since SP3 is a downstream target of *miR*-*506* and promotes migration and invasion of breast cancer cells, we sought to ask if *miR*-*506* regulates migration and invasion of breast cancer cell lines in an SP3-dependent manner. First, we confirmed that miR-506-regulated SP3, DNMT1 and MEG3 expression levels could be reverted by SP3 overexpression in mRNA level (Fig. 5a). Wound healing assays revealed that *miR*-*506* mimic significantly inhibited the migration of MCF-7 and MDA-MB-231 cells compared to NC mimic group (Fig. 5b, c). More importantly, we found that SP3 had the ability to largely rescue *miR*-*506* overexpression-impaired migration. Similarly, transfection of *miR*-*506* alone in MCF-7 or MDA-MB-231 cells led to decreased invasion ability compared to NC mimic cells; however, cells co-transfected with both *miR*-*506* and SP3 displayed invasion ability comparable to cells transfected with NC mimic (Fig. 5d, e).

## Discussion

The major finding of the present study is that *miR*-*506* inhibits migration and invasion of breast cancer cell lines through an undescribed pathway SP1/SP3/DNMT1/*MEG3*. We revealed a novel epigenetic mechanism of how *miR*-*506* and SP3 play a role in breast cancer progression.

Many studies have shown that *miR*-*506* functions as tumor suppressor in different types of malignant tumors [25, 26]. For instance, Chen et al. [27] reported that *miR*-*506* inhibits colorectal cancer progression by targeting DNMT1 and DNMT3b. In the present study, we further uncover SP1 and SP3 as novel targets by which *miR*-*506* regulates DNMT1 expression. A previous meta-analysis revealed that *miR*-*506* is associated with the survival of breast cancer patients [28]. Recently, *miR*-*506* has been shown to regulate TGFβ1-induced EMT of breast cancer cells through targeting EMT-related gene expression [11]. Although it has been found that *miR*-*506* has the ability to repress IQGAP1 and MAPK signaling pathway to influence breast cancer metastasis, other downstream targets may exist, which prompted us to search by bioinformatic prediction and for the first time, find out SP1 and SP3 as a direct target of *miR*-*506* [29], thereby regulating breast cancer metastasis via DNMT1/*MEG3* axis. SP1 and SP3 expression level are often greater in cancer cells than in normal cells [23]. Compared to SP3, SP1 has been extensively studied in breast cancer, thyroid cancer, hepatocellular cancer, pancreatic cancer, colorectal cancer, gastric cancer and lung cancer [30–32]. Hence, we primarily focused on the role of SP3 in present study, bridging *miR*-*506* and DNMT1/*MEG3*.

It was found that the demethylation of *MEG3* promoter and the change of gene region are the main reasons for the abnormal expression of *MEG3* in tumors [33]. Consistently, another study has shown that *MEG3* expression is closely regulated by DNA methylation with the treatment of DNA methylation inhibitor (5′-Aza-2′-deoxycytidine) [18]. The further work can be focused on assessing whether other members of DNMT (e.g., DNMT3a and DNMT3b) or DNA demethylases (e.g., TET1-3) are implicated in regulating the expression of *MEG3* mediated by miR-506. Besides, another interesting question is to search for other miRNAs responsible for upregulating *MEG3* through targeting DNMT1.

A number of previous studies have identified *MEG3* as a classical tumor suppressor [34, 35]. The mechanism of how *MEG3* exerts its effects on tumorigenesis is almost fully understood. To date, two groups have confirmed that *MEG3* suppresses tumorigenesis and progression of breast cancer and gliomas by p53 pathway [17, 18]. In addition, by comparing gene expression profiles in embryonic brains between *Meg3* KO mice and wild-type mice using microarray techniques, researchers found that *Meg3* deletion could lead to elevated VEGFA and VEGFR1 [36]. Based on these findings, we do not draw much attention on the downstream signaling regulated by *MEG3* or function of *MEG3.*

## Conclusion

In conclusion, our study highlighted a novel regulation axis responsible for *miR*-*506*-attenuated migration and invasion of breast cancer cell lines. Of which, for the first time, we reveal *miR*-*506* has a role in targeting and regulating SP1 and SP3 expression, down-regulating methylation level of MEG3 promoter in a DMNT1 dependent manner. Our findings provide new mechanism for explaining the breast cancer progression as well as potential candidate for treating breast cancer in future.

## Abbreviations

DNMT: DNA methyltransferase
SP/KLF: specificity protein/Kruppel-like factor
DMEM: Dulbecco’s Modified Eagle’s Medium
shRNAs: short hairpin RNAs
